# Macrophages of multiple hematopoietic origins reside in the developing prostate

**DOI:** 10.1242/dev.203070

**Published:** 2024-08-29

**Authors:** Sally W. Feng, Tanya M. North, Peri Wivell, Andrew Pletcher, Anastas Popratiloff, Maho Shibata

**Affiliations:** ^1^ Department of Anatomy and Cell Biology, The George Washington University School of Medicine and Health Sciences, Washington, DC 20052, USA; ^2^ The George Washington University Cancer Center, The George Washington University School of Medicine and Health Sciences, Washington, DC 20052, USA; ^3^ GW Nanofabrication and Imaging Center, The George Washington University, Washington, DC 20052, USA

**Keywords:** Prostate development, Macrophage, Hematopoietic origin, Puberty, Mouse

## Abstract

Tissue-resident macrophages contribute to the organogenesis of many tissues. Growth of the prostate is regulated by androgens during puberty, yet androgens are considered immune suppressive. In this study, we characterized the localization, androgen receptor expression and hematopoietic origin of prostate macrophages, and transiently ablated macrophages during postnatal prostate organogenesis in the mouse. We show that myeloid cells were abundant in the prostate during puberty. However, nuclear androgen receptor expression was not detected in most macrophages. We found *Cx3cr1*, a marker for macrophages, monocytes and dendritic cells, expressed in interstitial macrophages surrounding the prostate and associated with nerve fibers. Furthermore, we provide evidence for the co-existence of embryonic origin, self-renewing, tissue-resident macrophages and recruited macrophages of bone-marrow monocyte origin in the prostate during puberty. Our findings suggest that prostate macrophages promote neural patterning and may shed further light on our understanding of the role of the innate immune system in prostate pathology in response to inflammation and in cancer.

## Teaser

**Summary:** Macrophages are abundant in the developing mouse prostate during puberty, associating with nerve fibers and vasculature.

## INTRODUCTION

Prostate organogenesis starts before birth, with extensive tissue growth occurring during puberty in response to androgens (Pletcher and Shibata, 2022). Prostate organogenesis in rodents resembles that of humans. In mice, solid prostate epithelial buds emerge from the urogenital epithelium (UGE) at embryonic day (E) 17.5. The buds elongate and branch, eventually growing into canalized prostate ducts with differentiated luminal and basal epithelial cells (Shibata et al., 2020). Subsequently, androgens promote prostate growth during puberty (Tika et al., 2019). The development of the immune system begins during embryonic development, and in mice continues into the early postnatal period (Holsapple et al., 2003). Thus, the androgen-induced tissue growth occurs after the initial development of the immune system. Androgens are generally considered immune suppressive (Klein and Flanagan, 2016; Trigunaite et al., 2015), and an immunosuppressive microenvironment is needed as new prostate-specific proteins are generated during prostate organogenesis.

Macrophages are part of the innate immune system and can have specialized tissue-specific functions. Historically, tissue-resident macrophages were thought to originate from monocytes generated by bone-marrow hematopoietic stem cells after birth (Lee and Ginhoux, 2022; Mass et al., 2023). However, recent fate-mapping studies revealed that many adult tissue-resident macrophages arise during embryonic development from yolk sac erythro-myeloid progenitors (EMPs), which give rise to pre-macrophages that colonize embryonic tissues (Lazarov et al., 2023; Mass et al., 2023). Yolk sac EMPs begin developing at E7.5, whereas fetal liver monocytes differentiate from the EMPs that migrate to the fetal liver at E10.5 or develop locally from hematopoietic stem cell precursors (Henneke et al., 2021; Lee and Ginhoux, 2022). Tissue-resident macrophages from distinct macrophage precursors differ in gene expression profiles (van de Laar et al., 2016), and could have different functional capabilities.

The hematopoietic origin and functions of macrophages are organ specific, and the proportion of macrophages in an organ from distinct hematopoietic origins can shift throughout life (Lazarov et al., 2023; Mass et al., 2023). For example, in the mammary gland, ductal macrophages arise from embryonic precursors, and bone marrow-derived monocytes contribute to the rapid expansion of macrophages during puberty (Dawson et al., 2020). Macrophages of varying hematopoietic origins perform distinct tissue-specific functions in organogenesis and homeostasis, as described in organs such as the testes (Gu et al., 2023) and liver (Li et al., 2023). Tissue-resident macrophages are also essential for proper development and maintenance of innervation in the gut (Matheis et al., 2020; Viola et al., 2023) and brown adipose tissue (Wolf et al., 2017). How heterogeneous populations of macrophages regulate postnatal prostate organogenesis is not known and would significantly improve our understanding of macrophage function in prostate disease.

Single-cell RNA-sequencing studies of mouse and human adult prostates have revealed previously unknown heterogeneity of prostate luminal cell types conserved in both mouse and human adult prostates (Crowley et al., 2020; Guo et al., 2020; Henry et al., 2018; Karthaus et al., 2020). Additionally, these studies identified distinct clusters of immune cells, including monocytes, macrophages and T cells, in the prostate (Crowley et al., 2020; Guo et al., 2020; Karthaus et al., 2020). Notably, a prostate-specific macrophage population that maintains zinc levels in adult mouse and human prostates has also been identified (Tuong et al., 2021). However, the heterogeneity and specific function of these immune cell populations, and particularly their functions during prostate organogenesis, remain uncharacterized.

In this study, we conducted *in vivo* lineage-tracing studies to understand the localization, androgen dependence, cellular origin and function of macrophages during mouse prostate organogenesis. We provide evidence for embryonic and bone-marrow monocyte-derived tissue-resident prostate macrophages that expand during puberty.

## RESULTS

### Increase in myeloid cells in the prostate during puberty

**Fig. 1.**
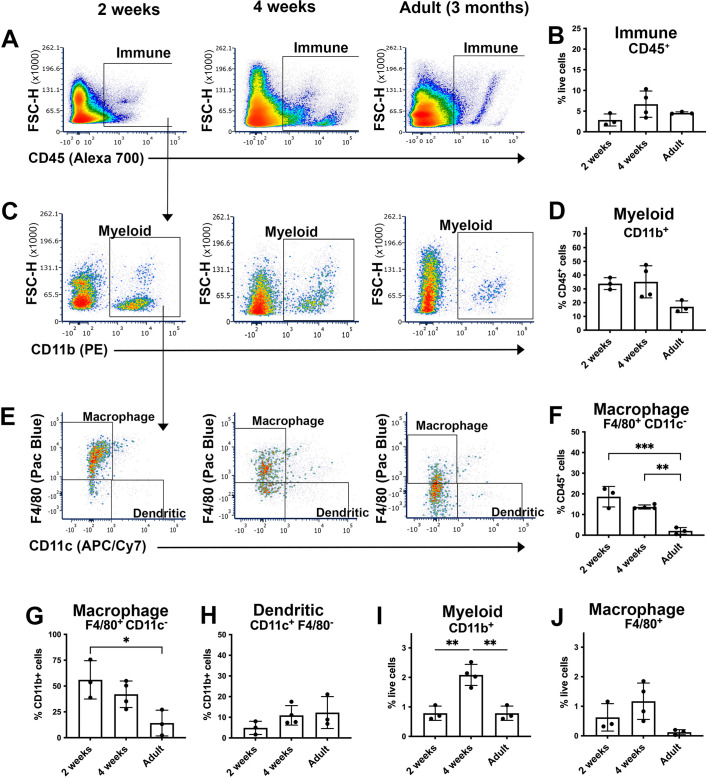
**Increase in myeloid cells in the mouse prostate during development.** (A-J) Flow cytometry analysis of wild-type C57BL/6 whole prostate tissues from mice at 2 weeks, 4 weeks and 3 months (adult) of age. Cells shown were gated on live single cells (Fig. S1B). (A,B) Representative plots showing gating of CD45^+^ immune cells and quantitation. (C,D) Representative plots showing gating of CD11b^+^ myeloid cells and quantitation. (E-H) Representative plots showing gating of F4/80^+^ CD11c^−^ macrophages and CD11c^+^ dendritic cells and quantitation. (I,J) CD11b^+^ myeloid and F4/80^+^ macrophage cell populations as a percentage of total live cells across the three age groups. Sample sizes were *n*=3 pools of 4 prostates at 2 weeks, *n*=4 pools of 2 prostates at 4 weeks and *n*=3 adult prostates. Error bars represent s.d. *P*-values were calculated using a one-way ANOVA test. *P<0.05; **P<0.01, ***P<0.001. APC, allophycocyanin; FSC-H, forward scatter height; PE, phycoerythrin.

Unbiased single-cell RNA-sequencing studies of the adult mouse prostate have revealed heterogeneous macrophage populations (Crowley et al., 2020; Guo et al., 2020; Karthaus et al., 2020; Tuong et al., 2021). To compare the abundance of macrophages at different stages of postnatal prostate organogenesis, we conducted flow cytometry analysis of dissociated cells from whole prostates collected from mice aged 2 weeks (prior to puberty), 4 weeks (puberty) and 3 months (adult) (Fig. 1, Fig. S1). The mean percentage of CD45 (Ptprc) positive (^+^) immune cells consisted of <7% of live cells and did not differ between the three time points (Fig. 1A,B). We then examined the proportion of CD11b (Itgam)^+^ myeloid cells during prostate organogenesis. The mean proportion of CD45^+^ cells that expressed CD11b ranged from 34% (s.d.±4%) at 2 weeks of age and 35% (s.d.±12%) at 4 weeks to 17% (s.d.±4%) in adults (Fig. 1C,D). We also found that CD45 expression was low in CD11b^+^ myeloid cells (Fig. S1C).

In prostates from 4-week-old mice, 42% (s.d.±12%) of CD11b^+^ myeloid cells expressed high or intermediate levels of the macrophage marker F4/80 (Adgre1) (Fig. 1E-G). Ly6G^+^ neutrophils and CD11c (Itgax)^+^ dendritic cells were less abundant compared with F4/80^+^ macrophages (Fig. 1H, Fig. S1D). The overall percentage of CD11b^+^ myeloid cells in the prostate decreased after puberty (Fig. 1I,J). Altogether, these results suggest that macrophages are more abundant in the prostate during postnatal prostate organogenesis (before and during puberty), compared with after puberty.

### Macrophages lacking nuclear AR expression are associated with prostate ducts

**Fig. 2.**
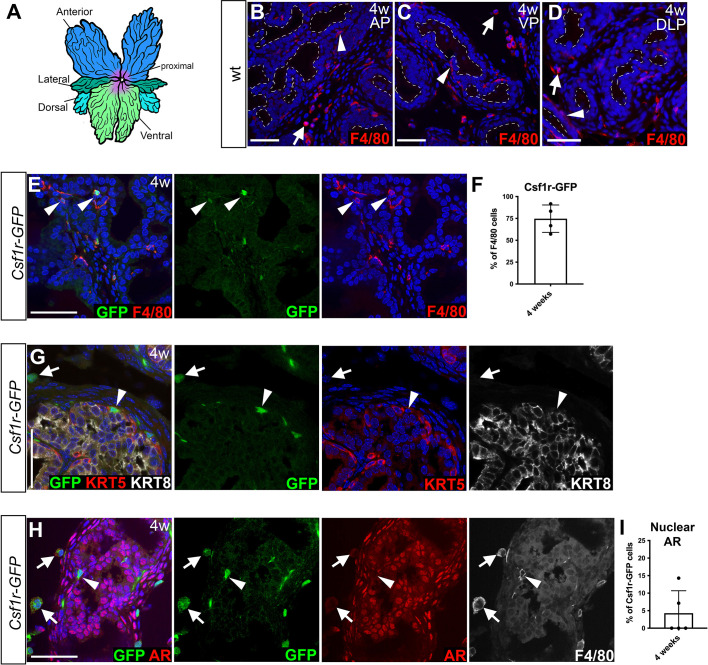
**Characterization of prostate macrophage localization during puberty.** (A) Illustration of a mouse prostate. (B-D) Analysis of F4/80 expression by immunostaining of anterior prostate (AP), ventral prostate (VP) and dorsal lateral prostate (DLP) from C57BL/6 mice at 4 weeks of age (4w). Dashed white lines outline ductal lumens. (E-H) *Csf1r-GFP* expression in the prostate. (E,F) Representative images and quantitation of immunofluorescence staining for GFP and F4/80 expression in AP tissues from Csf1r-GFP mice at 4 weeks of age (*n*=4 mice, 38 cells). (G) Immunostaining for GFP, the basal cell marker KRT5 and the luminal cell marker KRT8. (H) Immunostaining for GFP, AR and F4/80. (I) Quantitation of the staining shown in H (*n*=5 mice, 121 cells). Arrowheads point to ductal cells, and arrows point to interstitial cells expressing GFP (green). Nuclei were stained with DAPI. Scale bars: 50 µm.

To determine the localization of the CD11b^+^ myeloid cells and F4/80^+^ macrophage cells we identified by flow cytometry, we conducted immunofluorescence staining (Fig. 2, Fig. S2). We examined all prostate lobes and found F4/80^+^ macrophages in both the interstitial tissue (arrows in Fig. 2B-D, Fig. S2A) and associated with prostate ducts (arrowheads in Fig. 2B-D, Fig. S2A). Consistent with our findings from flow cytometry, we detected more CD11b^+^ and F4/80^+^ cells in prostates at 4 weeks of age compared with adult prostates, relative to the number of luminal cells (Fig. S2B-G). Additionally, more cells expressed the macrophage marker CD68 at 4 weeks of age compared with adult prostates (approximately 66% of CD11b^+^ cells), confirming findings from flow cytometry that many of the CD11b^+^ myeloid cells are likely macrophages (Fig. S2D).

To characterize these macrophages further, we focused on the anterior prostate lobe as this is the largest lobe, and used a transgenic *Csf1r-GFP* mouse line that expresses enhanced green fluorescent protein (GFP) in myeloid cells (Burnett et al., 2004; Sasmono et al., 2003). Csf1r-GFP cells were found in interstitial and ductal regions of the prostate (Fig. 2E-H), and 94.7% of Csf1r-GFP cells in the prostate expressed F4/80 at 4 weeks of age (Fig. 2E-H; *n*=4 mice, 38 cells), suggesting that most of the Csf1r-GFP^+^ cells in the prostate at this age were macrophages. Seventy-five percent (s.d.±16%) of F4/80^+^ cells in the prostate expressed Csf1r-GFP (Fig. 2F; *n*=4 mice). Co-staining for the basal cell marker keratin 5 (KRT5) and the luminal cell marker keratin 8 (KRT8) revealed Csf1r-GFP cells adjacent to both epithelial cell types (Fig. 2G). Of note, interstitial macrophages had a round or dendritic morphology, whereas ductal macrophages displayed a dendritic morphology (Fig. 2B-H).

Csf1r-GFP-expressing cells were detected at all stages of postnatal prostate organogenesis from postnatal day (P) 0 to 8 weeks of age (Fig. S2H-K). Given the increase in the proportion of CD11b^+^ myeloid cells during puberty (Fig. 1, Fig. S2), we investigated whether prostate macrophages directly respond to rising circulating androgen levels by assessing androgen receptor (AR) expression. Nuclear AR localization, indicative of active genomic AR signaling, was detected in luminal prostate epithelial cells in 4-week-old mice. Intriguingly, nuclear AR expression was not detected in 93.4% of Csf1r-GFP^+^ cells from 4-week-old prostates, and 89.7% of Csf1r-GFP^+^ cells from 8-week-old prostates (Fig. 2H,I: *n*=5 mice, 121 cells at 4 weeks; Fig. S2L,M: *n*=3 mice, 87 cells at 8 weeks). Altogether, these findings suggest that macrophages expressing F4/80 and *Csf1r*, but that lack nuclear AR, are present in interstitial and ductal areas of the prostate during puberty.

### *Cx3cr1*-expressing interstitial macrophages surround prostate ducts

To evaluate further the localization of macrophages during postnatal prostate organogenesis, we conducted 3D confocal imaging of whole prostate tissues. To facilitate 3D imaging, we used a bright enhanced YFP reporter (Madisen et al., 2010) to genetically label macrophages using an inducible *Cx3cr1^CreERT2^* allele (Madisen et al., 2010; Yona et al., 2013). *Cx3cr1* is expressed in mononuclear phagocytes (macrophages, monocytes and dendritic cells), although expression can be lost in some macrophage populations (Yona et al., 2013).

**Fig. 3.**
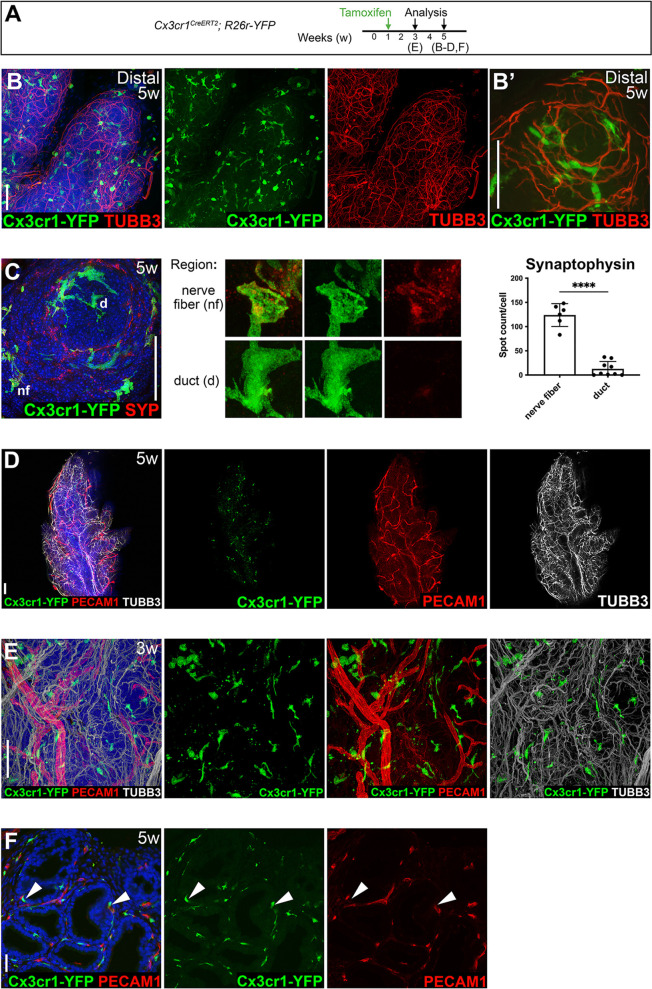
**3D confocal imaging of prostate macrophages reveals association with neurons and vasculature.** (A) Timeline for YFP labeling and analysis of anterior prostates from *Cx3cr1^CreERT2^; R26-YFP* (Cx3cr1-YFP) mice at 3-5 weeks of age. Letters indicate the ages of samples shown in subsequent panels. (B-E) 3D confocal (B-D) and two-photon imaging (E) of macrophages immunostained for the neuronal markers tubulin beta 3 class III (TUBB3; B,B′,D,E) and synaptophysin (SYP; C) reveals abundant Cx3cr1-YFP-expressing cells surrounding the surface of prostate ducts. B′ is a higher magnification region from B. Maximum intensity projections of *z*-stacks of confocal images of 50 µm (B,D), 15 µm (B′) and 20 µm (C) thickness, and a two-photon image of 35 µm thickness (E) are shown. (E) is related to Movie 3. (F) Confocal image from a tissue section stained for the endothelial cell marker PECAM1. Arrowheads indicate Cx3cr1-YFP-expressing cells. Sample sizes were at least *n*=3 mice for each staining. Sample size for quantitation of SYP staining was *n*=3 mice and 15 total cells. *P*-value was calculated using a two-tailed Student's *t*-test. ****P<0.0001. Nuclei were stained with DAPI. Scale bars: 50 µm.

We treated *Cx3cr1^CreERT2^; R26-YFP* males with tamoxifen at P7 to induce YFP expression and imaged Cx3cr1-YFP cells in prostate tissues (Fig. 3, Fig. S3). We confirmed that 91.8% of Cx3cr1-YFP cells co-expressed the macrophage marker F4/80 at 5 weeks of age (Fig. S3B). 3D volumetric confocal imaging revealed Cx3cr1-YFP cells in the prostate stroma surrounding the surface of prostate ducts (Fig. S3C). We immunostained and optically cleared wholemount prostate tissues with clearing-enhanced 3D plus (Ce3D+) (Anderson et al., 2020; Li et al., 2019) for 3D volumetric confocal imaging throughout the thickness of the anterior prostate (AP; approximately 200 µm for 5-week-old mice). To visualize the proximity of Cx3cr1-YFP cells to prostate ductal epithelial cells, we stained prostate tissues for the basal cell marker KRT5 (Fig. S3D, Movie 1). Many Cx3cr1-YFP cells were found in the interstitial tissue surrounding the prostate ducts, although Cx3cr1-YFP cells adjacent to basal cells, marked by KRT5 expression, and luminal cells, marked by KRT8 expression, were also observed (Fig. S3E).

Based on previous reports revealing the association of neurons and tissue-resident macrophages with high *Cx3cr1* expression (Chakarov et al., 2019), we assessed expression of a pan-neuronal marker, tubulin, beta 3 class III (TUBB3), and a synaptic vesicle marker, synaptophysin (SYP) (Fig. 3, Fig.S3F-H). 3D imaging revealed the proximity of Cx3cr1-YFP cells to nerve fibers, suggesting an association of macrophages with nerve fibers. These neuron-associated Cx3cr1-YFP cells and nerve fibers were found along the surface of the prostate ducts, and along the proximal-distal axis of the prostate lobe in both distal prostate tips (Fig. 3B,B′, Movie 2) and more proximal regions (Fig. S3G). We also observed an increase in synaptophysin-stained puncta in Cx3cr1-YFP cells associated with nerve fibers compared with Cx3cr1-YFP cells adjacent to ductal epithelial cells (Fig. 3C). Immunostaining for PECAM1, a marker of endothelial cells, additionally revealed a close association of Cx3cr1-YFP cells to prostate vasculature (Fig. 3D-F, Fig. S3H, Movie 3). These findings suggest that macrophages associated with nerve fibers may engulf neuronal material, similar to macrophages of the gut that remodel the enteric nervous system postnatally (Viola et al., 2023).

### Macrophages of embryonic and bone-marrow monocyte origin contribute to prostate organogenesis

**Fig. 4.**
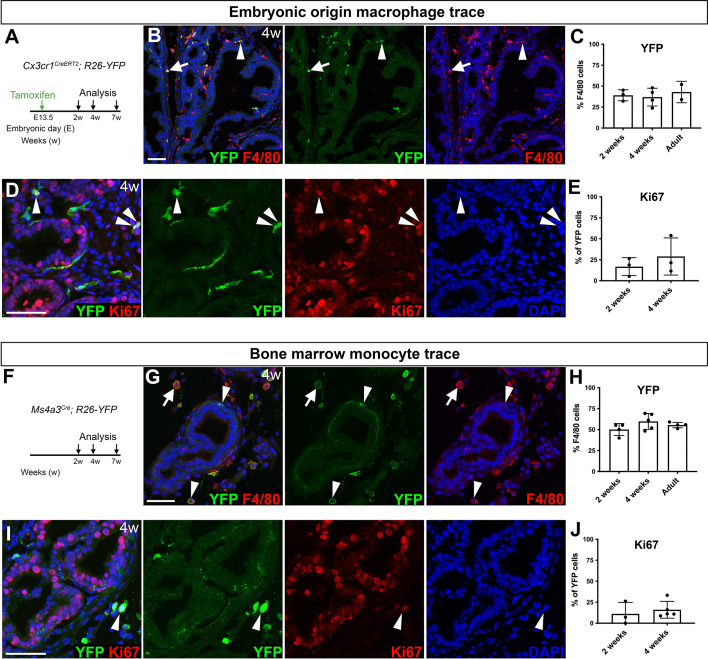
**Macrophages of heterogeneous hematopoietic origin in the developing prostate.** (A) Timeline for lineage labeling and tracing of macrophages of embryonic origin. (B-E) Analysis of embryonic origin macrophages in anterior prostates from *Cx3cr1^CreERT2^; R26-YFP* mice at 2 weeks, 4 weeks and adult (7-10 weeks) of age. Images show YFP and F4/80 expression (B) and YFP and Ki67 (D) expression at 4 weeks. Graphs show the percentage of F4/80^+^ cells expressing YFP (C) and the percentage of YFP^+^ cells expressing Ki67 (E). Total sample sizes were *n*=6 mice at 2 weeks, *n*=8 mice at 4 weeks and *n*=6 mice at 7-10 weeks. Arrowheads in D point to YFP^+^ Ki67^+^ cells. (F) Timeline for analysis of bone marrow-derived monocytes using *Ms4a3^Cre^; R26-YFP* mice. (G-J) Analysis of YFP and F4/80 expression (G,H), and analysis of YFP and Ki67 expression (I,J) in anterior prostates. Arrowheads in I point to a YFP^+^ Ki67^+^ cell. Total sample sizes for *Ms4a3^Cre^; R26-YFP* mice were *n*=3-4 mice at 2 weeks, *n*=5 mice at 4 weeks, and *n*=4 mice at 7 weeks. Arrowheads in B,G point to YFP^+^ F4/80^+^ ductal cells. Arrows in B,G point to YFP^+^ F4/80^+^ interstitial cells. Nuclei were stained with TO-PRO-3 in B, and DAPI in D,G,I. Scale bars: 50 µm.

To determine whether heterogeneous macrophage populations in the prostate have distinct cellular origins and functions, we performed genetic fate-mapping studies of embryonic origin and bone-marrow monocyte origin macrophages. We permanently labeled embryonic origin macrophages at E13.5 using *Cx3cr1^CreERT2^; R26-YFP* animals (Jappinen et al., 2019; Yona et al., 2013). We analyzed prostates at ages 2 weeks (prior to puberty; *n*=6 mice), 4 weeks (puberty; *n*=8 mice) and 7-10 weeks (adult; *n*=6 mice; Fig. 4A-E, Fig. S4).

Lineage tracing of embryonic origin macrophages revealed co-expression of YFP and the macrophage marker F4/80 in prostate ductal and interstitial regions (Fig. 4B, Fig. S4D,E). As a positive control, we analyzed microglia in the brain, which are of yolk sac origin (Fig. S4B,C) (Gomez Perdiguero et al., 2015). In tissue sections, the YFP lineage labeling efficiency of F4/80^+^ cells was approximately 60% in the brain cortex (Fig. S4B,C; *n*=4 mice, 171 cells). In the prostate, YFP labeling frequency of F4/80^+^ cells was 40% (Fig. 4C, Fig.S4D,E). We also assessed the YFP labeling frequency of CD11b^+^ myeloid cells in the prostate by flow cytometry at 7 weeks of age, when prostates were larger, but found that the percentage of YFP^+^ cells was lower, at 2% (Fig. S4F,G; *n*=4 mice).

To determine whether embryonic origin prostate macrophages undergo self-renewal, we assessed proliferation of lineage-traced macrophages by immunofluorescence staining for Ki67 (Mki67). During puberty, prostate epithelial cells are highly proliferative (Tika et al., 2019) (Fig. 4D). We detected YFP^+^ embryonic-derived macrophages that expressed Ki67, suggesting that proliferation of embryonic-derived macrophages could contribute to the increase of macrophages during puberty (Fig. 4D,E).

We also examined CD74 and LYVE1 expression in embryonic origin lineage-traced cells in the prostate, as these proteins have previously been identified as markers for potentially distinct populations of macrophages in murine lung and mammary gland and human prostate tissues (Chakarov et al., 2019; Tuong et al., 2021; Wang et al., 2020). We observed both CD74^+^ and LYVE1^+^ populations in the embryonic origin lineage-traced macrophages (Fig. S4H). CD74^+^ cells were found near prostate ducts and interstitial areas, whereas LYVE1^+^ cells were found almost exclusively in interstitial areas (Fig. S4H). Altogether, these findings suggest that heterogeneous subtypes of macrophages of embryonic origin are present during postnatal prostate organogenesis.

To assess whether prostate macrophages of bone-marrow-derived monocyte origin are recruited to the prostate, we conducted lineage tracing using *Ms4a3^Cre^; R26-YFP* (Ms4a3-YFP) mice, which express YFP in bone-marrow monocytes and their progeny (Liu et al., 2019). In prostates at 4 weeks, we found that Ms4a3-YFP macrophages expressed F4/80, displayed a round morphology and were found in prostate interstitial areas, although we also observed Ms4a3-YFP^+^ cells that were elongated and near the surface of the prostate ducts (Fig. 4F-H, Fig. S5). We detected Ms4a3-YFP cells expressing Ki67 and phospho-histone H3 (PHH3), suggesting that, like macrophages of embryonic origin, macrophages originating from bone-marrow monocytes are also capable of self-renewal in the prostate (Fig. 4I,J, Fig. S5F). The YFP labeling efficiency of CD11b^+^ CD172a (Sirpa)^+^ monocytes in Ms4a3-YFP mice was ∼80% in the blood (Fig. S5B). In the prostate, YFP expression was detected in 19% of CD11b^+^ myeloid cells (Fig. S5E). Heterogenous Ms4a3-YFP cells with high expression of either CD74 or LYVE1 were also detected (Fig. S5G). Altogether, these findings indicate the co-existence of tissue-resident macrophages of embryonic origin and bone-marrow monocyte-derived macrophages in the prostate during postnatal prostate organogenesis.

### Ablation of prostate macrophages affects nerve fiber tortuosity

To assess the function of macrophages during puberty, we generated *Cx3cr1^CreERT2/+^; R26-DTA/YFP* animals expressing diphtheria toxin in *Cx3cr1-*expressing cells (Cx3cr1-DTA) to ablate macrophages through apoptotic cell death (Voehringer et al., 2008). As a negative control, we generated *Cx3cr1^CreERT2^; R26-YFP* animals (Cx3cr1-YFP).

**Fig. 5.**
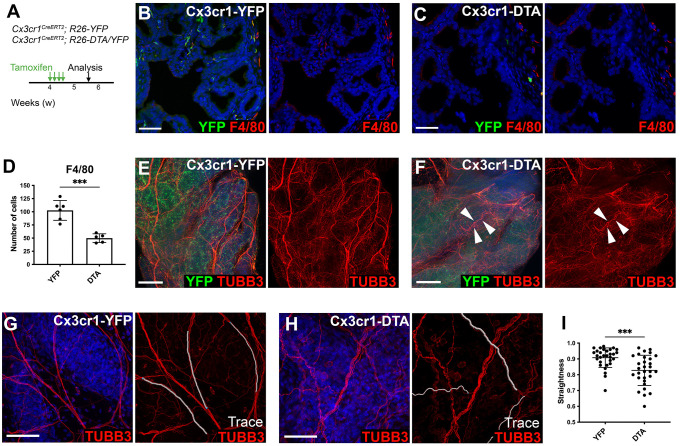
**Ablation of prostate macrophages affects nerve fibers.** (A) Timeline for analysis of anterior prostates from *Cx3cr1^CreERT2^; R26-YFP* (Cx3cr1-YFP) and *Cx3cr1^CreERT2^; R26-DTA/YFP* (Cx3cr1-DTA) mice. (B-D) Immunostaining of tissue sections for YFP and F4/80 (B,C) and quantitation (D; *n*=5-6 mice for each genotype). (E,F) Maximum intensity projections of 3D whole-mount confocal imaging for YFP and TUBB3. Arrowheads point to tortuous nerve fibers in Cx3cr1-DTA prostates. *z*-stacks were 125 µm. (G,H) 3D confocal imaging for TUBB3, with tracing of nerve fibers (white) using Imaris. (I) Quantification of nerve fiber tortuosity. A value of 1 indicates a straight fiber. Each dot in I represents a traced nerve fiber (*n*=10 nerve fibers each from 3-4 mice per genotype). Sample sizes for each staining were *n*>3 mice per genotype. Nuclei were stained with DAPI. Scale bars: 50 µm. *P*-values were calculated using two-tailed Student's *t*-tests. ***P<0.001.

We treated 4-week-old males with tamoxifen to induce Cre activation during puberty and confirmed YFP expression in F4/80-expressing macrophages (Fig. S6A,B). This time point was selected to reduce the effects of tamoxifen on early prostate organogenesis (Shibata et al., 2020). Shortly after Cre activation, we confirmed expression of the apoptotic marker caspase 3 (CASP3) in YFP^+^ cells from Cx3cr1-DTA cells, although we also observed CASP3 expression in control Cx3cr1-YFP cells (Fig. S6C-E). One week after Cre activation, Cx3cr1*-*YFP and F4/80 expression was reduced in Cx3cr1-DTA prostates compared with Cx3cr1-YFP prostates (Fig. 5A-D), confirming that macrophages were partially ablated in Cx3cr1-DTA prostates.

Wholemount TUBB3 staining and 3D imaging revealed growth of the prostate neural network during postnatal prostate organogenesis, with increased bundling and branching of axons (Fig. S6N). To assess the effects of macrophage ablation on nerves in the prostate, we examined nerve fibers after macrophage depletion. We compared Cx3cr1-DTA animals with control Cx3cr1-YFP animals and found that the neuronal network was more disorganized in Cx3cr1-DTA prostates compared with Cx3cr1-YFP control prostates (Fig. 5E-I). Cx3cr1-DTA prostates contained nerve bundles with increased tortuosity (arrowheads in Fig. 5F), whereas control Cx3Cr1-YFP prostates had straighter nerve bundles with a smoother appearance (Fig. 5G-I).

To confirm our observations on the changes in neuronal patterning upon macrophage ablation, we also ablated macrophages using transgenic macrophage Fas-induced apoptosis (MaFIA) mice (*Csf1r-FKBP1*) (Burnett et al., 2004). Prostate innervation begins prior to prostate bud formation and peaks at neonatal stages, with axon density increasing through P9 (Turco et al., 2019). Thus, we treated mice at 1 week of age with the dimerizer AP20187 to induce macrophage apoptosis at an earlier stage of prostate organogenesis (Fig. S6O-R). Upon partial ablation of macrophages, we observed nerve fibers with increased tortuosity, similar to Cx3cr1-DTA prostates, whereas nerve fibers were straighter and had a smoother morphology in untreated control prostate tissues (Fig. S6S-W).

Despite the reduction of YFP^+^ macrophages by tamoxifen induction of DTA in Cx3cr1-DTA prostates, F4/80^+^ macrophages were still detected after four consecutive days of tamoxifen treatment (Fig. 5B-D). Furthermore, the overall histological appearance of prostate tissues was normal in Cx3cr1-DTA prostates (Fig. S6F,G). To assess whether this may be due to proliferation of macrophages that escaped ablation, we conducted immunostaining for the macrophage marker CD68, YFP and the proliferation markers Ki67 and PHH3 (Fig. S6H-M). We detected Ki67 and PHH3 expression in CD68^+^ macrophages and YFP^+^ cells in both Cx3cr1-DTA and control Cx3cr1-YFP prostates, suggesting that proliferation of remaining macrophages, in addition to recruitment of macrophages, may contribute to the replenishment of ablated cells. Altogether, these macrophage-ablation experiments suggest that prostate macrophages interact with nerve fibers and regulate the innervation of the prostate during organogenesis.

## DISCUSSION

**Fig. 6.**
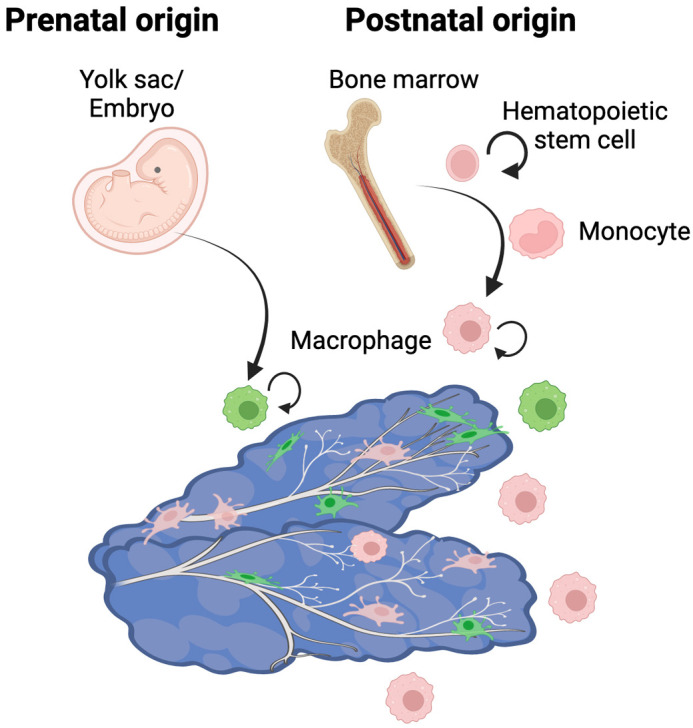
**Model for origin and function of macrophages in prostate organogenesis.** *Cx3cr1*-expressing macrophages surround the prostate and are associated with nerve fibers (depicted in pale gray). Both embryonic origin macrophages (depicted in green) and bone-marrow monocyte-derived macrophages (pink) contribute to prostate organogenesis and can self-renew in the prostate. Created with BioRender.com.

In this study, we investigated macrophage localization and function during prostate postnatal organogenesis in the mouse. We found that myeloid cells were abundant in the prostate during puberty, yet nuclear AR expression was not detected in most macrophages. We show that interstitial macrophages surrounding the prostate are associated with nerve fibers, and provide evidence for self-renewing tissue-resident macrophages from both embryonic origin macrophages and bone marrow-derived monocytes in the prostate (Fig. 6). Partial ablation of macrophages during prostate organogenesis affected prostate nerve fiber tortuosity.

The innate immune system develops before the androgen-driven growth in the prostate during puberty (Henneke et al., 2021). Androgens are generally thought to repress the immune system (Klein and Flanagan, 2016; Trigunaite et al., 2015). A prior study supporting this used LyzCre mice to knock out AR in myeloid cells, which resulted in increased cutaneous wound healing due to an enhanced inflammatory response (Lai et al., 2009). Thus, we assessed how puberty affects macrophages in the developing prostate. Despite the increase in macrophages in the prostate during puberty, the absence of nuclear AR expression in most prostate macrophages is intriguing, because nuclear AR expression is detected in most prostate epithelial cells and many stromal cells during puberty (Shibata et al., 2020). Consistent with our findings, previous single-cell RNA-sequencing experiments at E17.5 suggest that leukocytes in the developing urogenital sinus express low levels of *Ar* transcript (Lee et al., 2021). This suggests a possible non-cell-autonomous effect of androgens during puberty on macrophages in the prostate. However, other studies conducted in cell culture using bone marrow-derived macrophages or peripheral blood-derived macrophage cell lines suggest that AR is expressed and functional in adult prostate macrophages (Cioni et al., 2020). Together, these studies reveal context-specific heterogeneity or plasticity of macrophages in prostate tissues.

Our findings through high-resolution 3D imaging revealed that macrophages are abundant during prostate organogenesis and associated with the developing neural network. Neurons regulate growth and lumen formation in the developing salivary gland (Nedvetsky et al., 2014) and promote wound repair in the skin (Sebastian et al., 2017). In mouse models of prostate cancer, nerve endings have been shown to regulate angiogenesis and tumor growth (Zahalka et al., 2017). Similarly, nerve fibers regulate growth and homeostasis of the rat prostate (McVary et al., 1994; Wang et al., 1991). Thus, prostate innervation is also important for tissue growth. Although the development of autonomic and sensory axons in the prostate has previously been characterized (Turco et al., 2019), our studies provide a 3D visualization of the relationship between macrophages and nerve fibers and suggest a function for macrophages during prostate innervation. Although further studies are needed to understand the effect of macrophage ablation on neural function in the prostate, previous studies on peripheral nerve repair and gut homeostasis suggest that macrophages may play a role in maintaining neuronal function (De Schepper et al., 2018; Viola et al., 2023; Zigmond and Echevarria, 2019).

Through lineage tracing, we provide evidence for embryonic origin macrophages in the prostate that remain after puberty. We found that these embryonic origin macrophages can also proliferate in the prostate. Previous embryonic lineage-tracing studies suggest that yolk sac origin macrophages are labeled using the *Cx3cr1^CreERT2^* allele, as *Cx3cr1* is not expressed in fetal liver-derived monocytes (Jappinen et al., 2019; Madisen et al., 2010; Yona et al., 2013). However, we cannot exclude the possibility that fetal liver monocytes recruited to the prostate were lineage labeled after regaining *Cx3cr1* expression. We also did not directly examine the contribution of macrophages from fetal-liver origin monocytes to the prostate. By conducting additional lineage tracing of bone-marrow monocytes, we also identified bone marrow-derived macrophages in the prostate during postnatal development. Our findings suggest that these bone marrow-derived macrophages are mostly recruited to the prostate, but can also proliferate in the prostate. Our experimental findings confirm predictions from transcriptional profiles of macrophages in the adult prostate, which suggest both embryonic origin and bone-marrow monocyte origin macrophages may exist in the adult prostate (Tuong et al., 2021). Recruitment of bone marrow-derived macrophages to the prostate during puberty may resemble the mammary gland, where embryonic precursor ductal macrophages are gradually replaced by bone marrow-derived macrophages during puberty (Dawson et al., 2020).

Further research is needed to understand whether prostate macrophages from heterogenous precursors have distinct developmental functions. One limitation of this study was our use of F4/80 coupled to Pacific Blue, which limited our ability to detect F4/80 low cells and monitor changes in F4/80 expression levels in the prostate using flow cytometry. Use of a brighter fluorophore may improve the ability to distinguish F4/80 low cells (Liu et al., 2020). Additional work to extend these findings and compare macrophage functions during prostate organogenesis to functions in prostate diseases, including prostate cancer and benign prostatic hyperplasia, will be informative for developing new treatment approaches.

## MATERIALS AND METHODS

### Mouse strains

*Mus musculus* strains were analyzed on a C57BL/6J genetic background. *Csfr1-GFP* animals (MaFIA, JAX stock 005070) (Burnett et al., 2004) expressing EGFP in myeloid cells were obtained from The Jackson Laboratory and bred to C57BL/6J mice (JAX stock 000664). These animals also express dimerizer-inducible mutant human FK506 binding protein 1A, 12 kDa (FKBP12), which allows for inducible apoptosis of *Csf1r*-expressing cells. *Cx3cr1^CreERT2^* (JAX Stock 020940; Yona et al., 2013) and *Ms4a3^Cre^* (JAX stock 036382) (Liu et al., 2019) mice were obtained from The Jackson Laboratory and bred to *R26-YFP* (JAX Stock 007903; Madisen et al., 2010) mice to generate *Cx3cr1^CreERT2/+^; R26-YFP/+* and *Ms4a3^Cre/+^; R26-YFP/+* animals. *Cx3cr1^CreERT2/+^; R26-DTA/YFP* animals were generated by breeding to ROSA-DTA animals (JAX stock 009669; Voehringer et al., 2008). Animals were genotyped using primers from The Jackson Laboratory (Table S1). Swiss Webster females (Taconic, SW) were obtained from Taconic Biosciences and used as foster mice. Mice were socially housed with a 12 h light/12 h dark cycle, 18-24°C temperature, and 40-60% humidity-controlled specific pathogen-free (SPF) facility. All animal experiments were approved by the IACUC at the George Washington University.

### Tamoxifen treatment

Tamoxifen (Sigma-Aldrich, T5648) was dissolved in corn oil (Sigma-Aldrich, C8267) to generate a 20 mg/ml stock. For tamoxifen treatment of P7 male pups, a single subcutaneous injection of 50 µL of 5 mg/ml tamoxifen in corn oil was injected subcutaneously. For tamoxifen treatment of 4-week-old males, four intraperitoneal injections of tamoxifen, 200 mg/kg body weight, were given at P28, P29, P30 and P31. For Movie 2, Cx3cr1-YFP labeling was induced by intraperitoneal injections of tamoxifen at P26, P29 and P33, followed by tissue collection at P35.

### AP20187 dimerizer treatment

A stock solution of the dimerizer AP20187 was prepared by dissolving 1 mg AP20187 (Selleckchem, S8487) in 64 µl 100% ethanol. The dosing solution was prepared to a final concentration of 0.625 mg/ml AP20187 in 4% ethanol, 10% PEG-400, and 1.7% Tween 20 in water. At P7, P8 and P9, 50 µl AP20187 dosing solution was injected subcutaneously.

### Embryonic lineage tracing

Timed matings were set up to detect copulatory plugs at 0.5 days post-coitum. Pregnant mothers were treated with 1.5 mg tamoxifen dissolved in corn oil at 10 mg/ml via oral gavage at E13.5 (Jappinen et al., 2019; Yona et al., 2013). A solution containing 0.75 mg dose of progesterone (Sigma-Aldrich, P0130), prepared by diluting a 100 mg/ml ethanol stock with corn oil, was injected subcutaneously to counteract the tamoxifen. Pups were delivered by cesarean section at E18.5 or E19.5 and fostered by C57BL/6J or Swiss Webster foster mothers with litters born within one day.

### Tissue dissociation and flow cytometry

Animals were anesthetized with isoflurane and euthanized by cervical dislocation. Whole prostates were dissected and rinsed in cold 1× PBS and placed in 2 ml microcentrifuge tubes containing freshly prepared Digest Solution (Liu et al., 2020) comprising RPMI (ATCC) with 10% fetal bovine serum (Gibco, 26140079), 0.2 mg/ml Collagenase IV (Sigma-Aldrich, C5138) and 0.05 mg/ml DNase I (Roche, 10104159001). Either individual whole prostates or pooled prostates (pools of *n*=4 prostates at 2 weeks of age; *n*=2 prostates at 4 weeks of age) were minced with dissection scissors and incubated in a 6-well plate for 1 h at 37°C with shaking at 20 rpm using a VorTemp 56 incubator. Dissociation of the digested tissue was completed by mechanical shearing using a 16-gauge needle. The resulting suspension was passed through a 70 µm cell strainer (Fisherbrand) to obtain single/near-single cells and washed with cold PBS and spun down. Blood was collected after euthanasia by cardiac puncture using a 21.5 G hypodermic needle pre-rinsed with 0.5 M EDTA. The blood was transferred to an anticoagulant blood collection tube and red blood cells were lysed using Ammonium-Chloride-Potassium (ACK) lysis buffer as previously described (Liu et al., 2020). All subsequent washes/incubations were completed in FACs Buffer with 0.5% fetal bovine serum, 2 mM EDTA in PBS. Centrifugation was at 375 ***g*** for 5 min at 4°C. Cells were resuspended and manually counted using a hemacytometer. Cells were resuspended at 500,000 cells/100 µl and incubated with antibodies (Table S2) for 25 min at 4°C (Yende et al., 2023). After incubation, cells were washed, centrifuged at 375 ***g*** for 5 min and resuspended in FACs Buffer for flow cytometry. Analysis was performed on a BD Celesta analyzer with BD FACSDIVA and FCS Express 7 software (DeNovo Software). For each sample, 300,000-1,000,000 live cells were analyzed. Fluorescence Minus One (FMO) controls were used to gate cells.

### Tissue dissections and staining

Mouse tissues were dissected in 1× PBS. For cryosectioning, tissues were fixed in 4% paraformaldehyde at 4°C overnight, then placed in 30% sucrose solution. Tissues were frozen in Tissue-Tek Optimum Cutting Temperature (OCT) compound and 8-µm-thick sections were cut. For paraffin sectioning, prostate tissues were fixed in 10% formalin overnight. Tissues were processed using standard protocols and 5-µm-thick sections were cut. For staining of paraffin sections, antigen-retrieval was conducted using citrate-based antigen unmasking solution (Vector Laboratories, HH3301). Sections were blocked with 10% goat serum for 1 h at room temperature and primary antibodies were incubated overnight at 4°C. Primary antibodies used are listed in Table S2. Secondary fluorescence-conjugated antibodies from Invitrogen and Biotium were used (Table S2). Nuclei were counterstained with 4′,6-diamidino-2-phenylindole dihydrochloride (DAPI) or TO-PRO-3 (Invitrogen) prior to mounting in 90% glycerol with n-propyl gallate for microscopy analysis. For immunohistochemistry, secondary biotinylated antibodies and Vectastain Elite ABC HRP kit with NovaRED substrate from Vector Laboratories were used. Hematoxylin and Eosin staining was performed using a standard protocol using Gill 2 Hematoxylin. Confocal imaging was conducted using Zeiss Cell Observer spinning disk confocal, Leica TCS SP8 confocal and Zeiss 980 confocal microscopes.

### Wholemount staining, tissue clearing and 3D imaging

APs were dissected in 1× PBS. For prostates from mice 4 weeks and older, the lobes of the AP were split, and one half was used for whole-mount staining. The tissue was fixed overnight in 4% paraformaldehyde at 4°C, then permeabilized with 2% Triton X-100 in 1× PBS for 3 h on a rocker. All subsequent incubations were performed on a rocker at room temperature. The tissue was incubated with blocking buffer (10% fetal bovine serum, 0.02% sodium azide, 1% bovine serum albumin, 5% DMSO and 0.2% Triton X-100 in 1× PBS) for 3 h. Tissue was then incubated overnight with primary antibody and washed in wash buffer (5% DMSO, 0.2% Triton X-100 in 1× PBS) before staining with secondary antibody overnight. Tissues were incubated with 5 µg/ml DAPI at 1:1000 dilution in wash buffer for 3 h, then tissue was cleared in Ce3D+ (Anderson et al., 2020; Li et al., 2019). Ce3D+ solution was prepared in advance in a 50 ml conical tube by adding 20 g Histodenz^TM^ (final concentration 86% w/v; Sigma-Aldrich, D2158), 10.5 g of 40% N-methylacetamide in 1× PBS (final concentration 22% w/v; Sigma-Aldrich, M26305) and 22.5 mg Triton-X-100 (final concentration 0.1% w/v) and mixed overnight on a rotator (Anderson et al., 2021 preprint). Stained tissue was incubated in Ce3D+ overnight, then cleared tissue was mounted onto a glass slide in the same Ce3D+ solution (refractive index ND=1.515) (Anderson et al., 2020). A silicon spacer was placed around the tissue in accordance with tissue thickness (200 µm spacer for 200 µm thick tissue), and the mounted tissue was sealed with a glass coverslip on top with nail polish along the edges. Images were acquired on Zeiss Cell Observer spinning disk and Zeiss 980 confocal microscopes. *z*-stack images were acquired starting from the surface of the tissue. High-resolution images of TUBB3-labeled nerve fibers and SYP-positive puncta were acquired using Airyscan imaging on the Zeiss 980 confocal microscope. Movie 3 was acquired by two-photon spectral imaging on the Zeiss 980 confocal microscope using 920 nm laser excitation. Fluorophore emission curves were generated by probing a 32-channel lambda stack image set of the sample, and online linear spectral unmixing was conducted using Zen Software (Zeiss) to generate the spectral image.

### Image analysis

For quantitation of immunostaining, cells were manually counted using the ImageJ Cell Counter tool. Luminal ductal cells were identified by DAPI staining and manually counted based on nuclear size and morphology. Imaris 10 software (Oxford Instruments) was used for 3D visualization and surface tracing of acquired *z*-stacks of prostate tissue. For high-resolution images of a large field, tiled scans were stitched using Zen Software. Maximum intensity *z*-projections were generated using ImageJ version 2.14.0/1.54f. For Imaris quantitation of synaptophysin puncta engulfment by macrophages, macrophage cell volume was masked based on YFP fluorescence, and the number of puncta was counted using the Spots tool. TUBB3-labeled nerve axon fibers were manually traced using the Imaris Filament Tracer tool with semi-automatic tracing. Ten nerve segments were randomly selected from each prostate image. Segment straightness (defined as the ratio between the length of the fiber and the radial distance between the two anchor points) was computed using Imaris. Adobe Photoshop, BioRender, and Procreate were used to generate figures and illustrations.

### Statistical analysis

Sample sizes are stated in the figure legends, and no samples were excluded. Animals were allocated to experimental groups based on genotype. Normal distribution and variation were assumed. Statistical analyses (two-tailed Student's *t*-tests and one-way ANOVA) and graph generation were conducted using GraphPad Prism 10. Individual data points are plotted for small sample sizes (*n*<5).

## Supplementary Material

10.1242/develop.203070_sup1 Supplementary information
