# Supplementary material for: Macrophages of multiple hematopoietic origins reside in the developing prostate
Source: Development. 2024 Aug 29;151(16):dev203070. doi: 10.1242/dev.203070 (PMC11385323; doi:10.1242/dev.203070)
Supplement: Supplementary information [file develop-151-203070-s1.pdf]

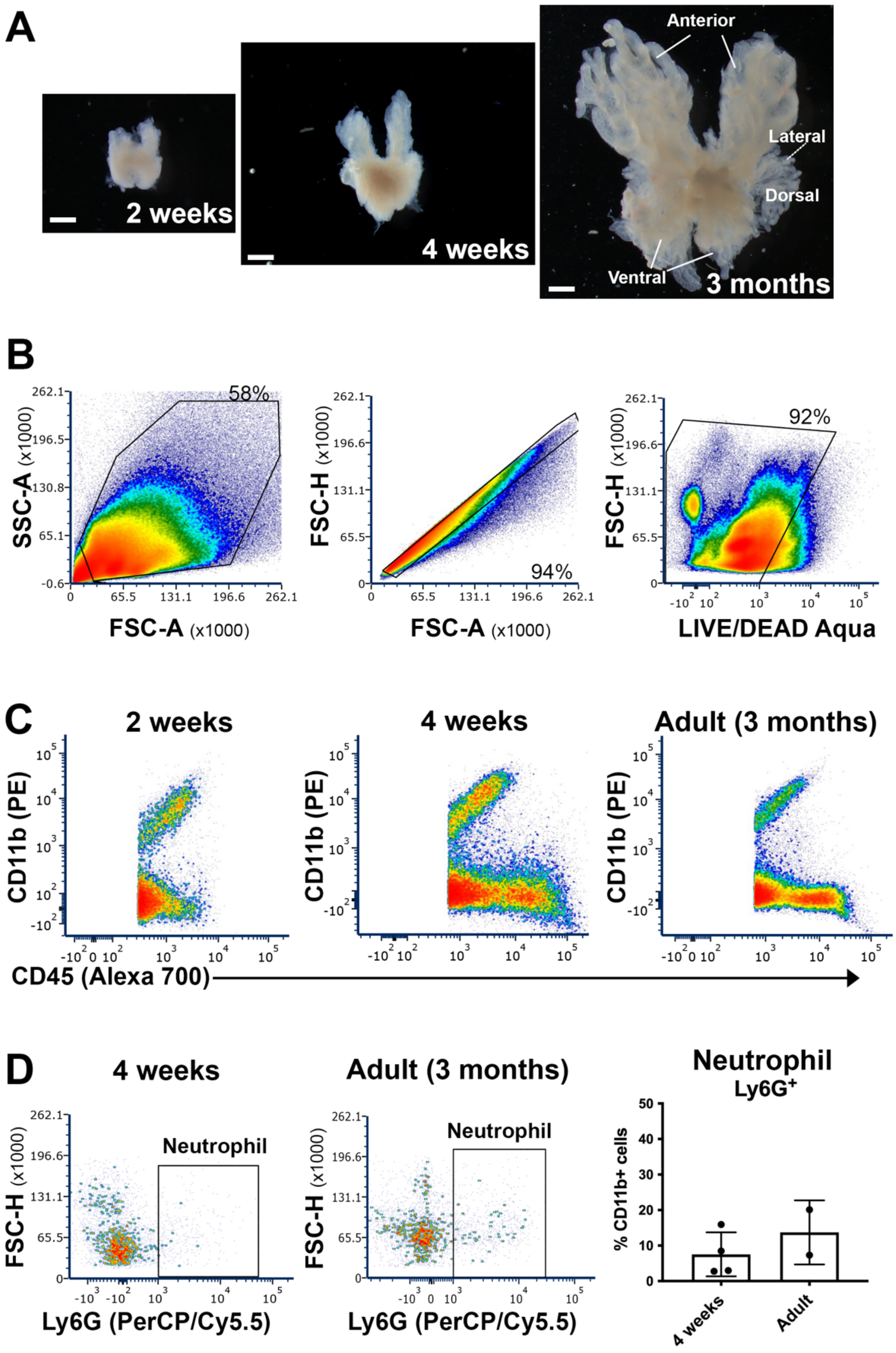

Development • Supplementary information

Fig. S1. Prostate dissections and flow cytometry gating strategy for identifying live single cells. (A) Whole-mount prostate images from mice at ages 2 weeks, 4 weeks and 3 months (adult). Scale bars indicate 1mm. (B) A representative flow cytometry plot for gating live single cells is shown. (C) Representative plots showing CD45 and CD11b expression after gating for CD45 as shown in Figure 1A-C. Flow cytometry analysis was conducted on wild type C57BL/6 whole prostate tissues from mice at 2 weeks, 4 weeks, and 3 months (adult) age and gated on live single cells. (D) Ly6G+ neutrophils (gated from live single CD45+ CD11b+ cells) in prostates from mice aged 4 weeks and 3 months. Sample sizes were n=4 pools of 2 prostates at 4 weeks, n=2 adult prostates. FSC-A, forward scatter area; FSC-H, forward scatter height; SSC-A, side scatter area. Related to Fig. 1.

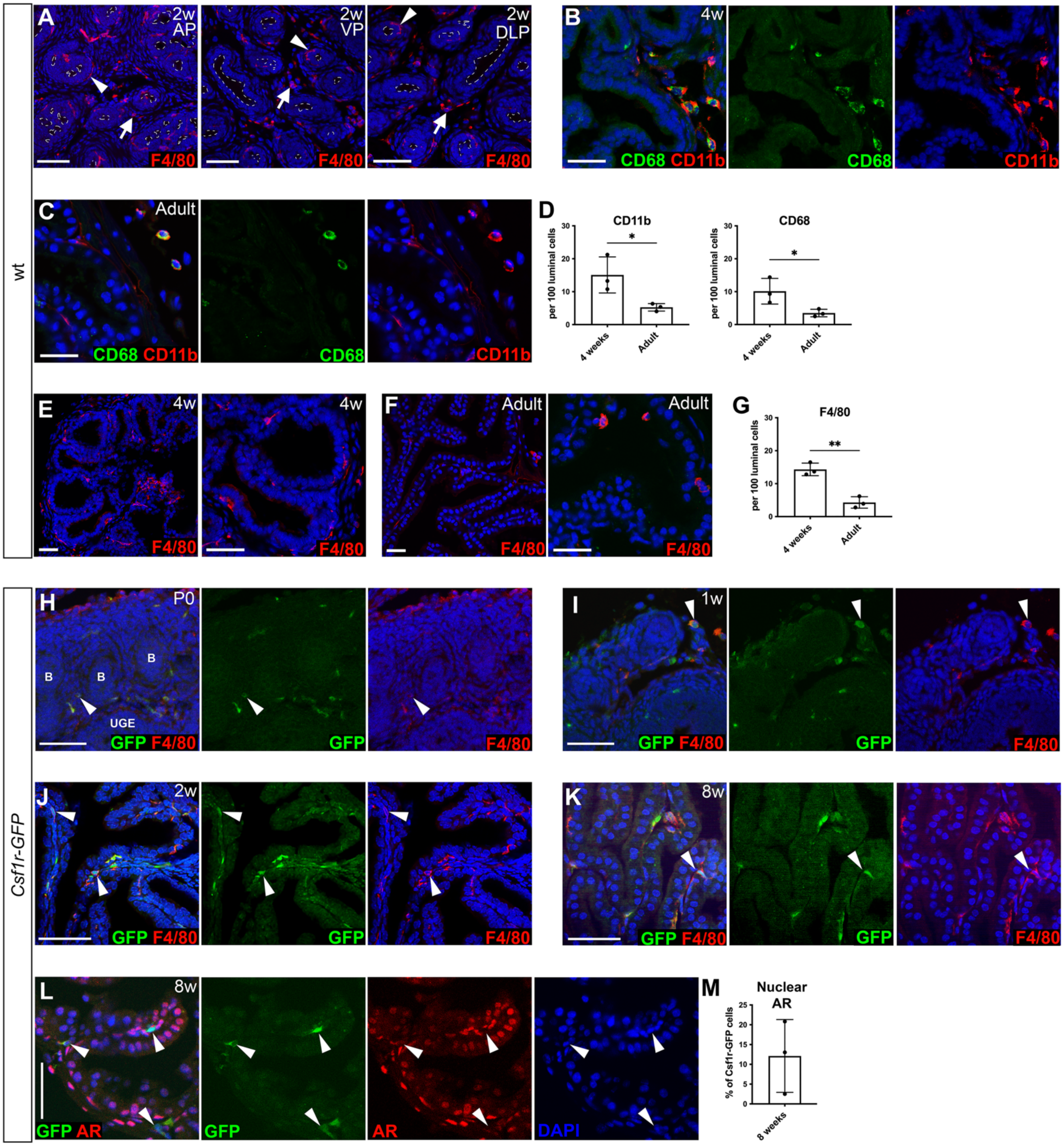

Development • Supplementary information

Fig. S2. Analysis of macrophage markers during prostate organogenesis. (A-G) Analysis and quantitation of (A, E-G) F4/80 and (B-D) CD68 and CD11b expression by immunostaining in wild-type C57BL/6 mouse prostates at 2 weeks, 4 weeks, and adults. Sample sizes were n=3 mice at each age. Broken white lines in A outline ductal lumens. (H-K) Analysis of tissue sections from *Csf1r-GFP* mice for expression of GFP and F4/80 at (H) postnatal day (P)0, (I) 1 week, (J) 2 weeks, and (K) 8 weeks of age. (L) Immunofluorescence staining for GFP and AR at prostates at 8 weeks in *Csf1r-GFP* mice and (M) quantitation (n=3 mice, 87 cells). Sample sizes for *Csf1r-EGFP* staining were n=5 at P0, n=4 at 1 week, n=2 at 2 weeks, n=4 at 4 weeks, and n=3 at 8 weeks. Anterior prostates are shown unless otherwise noted. Nuclei were stained with DAPI. Scale bars indicate 50 µm. *P* values were calculated using two-tailed student t-tests. *$p < 0.05$, **$p < 0.01$. AP, anterior prostate; VP, ventral prostate; DLP, dorsal-lateral prostate; B, prostate bud; UGE, urogenital epithelium; w, weeks; wt, wildtype. Related to Fig. 1 and Fig. 2.

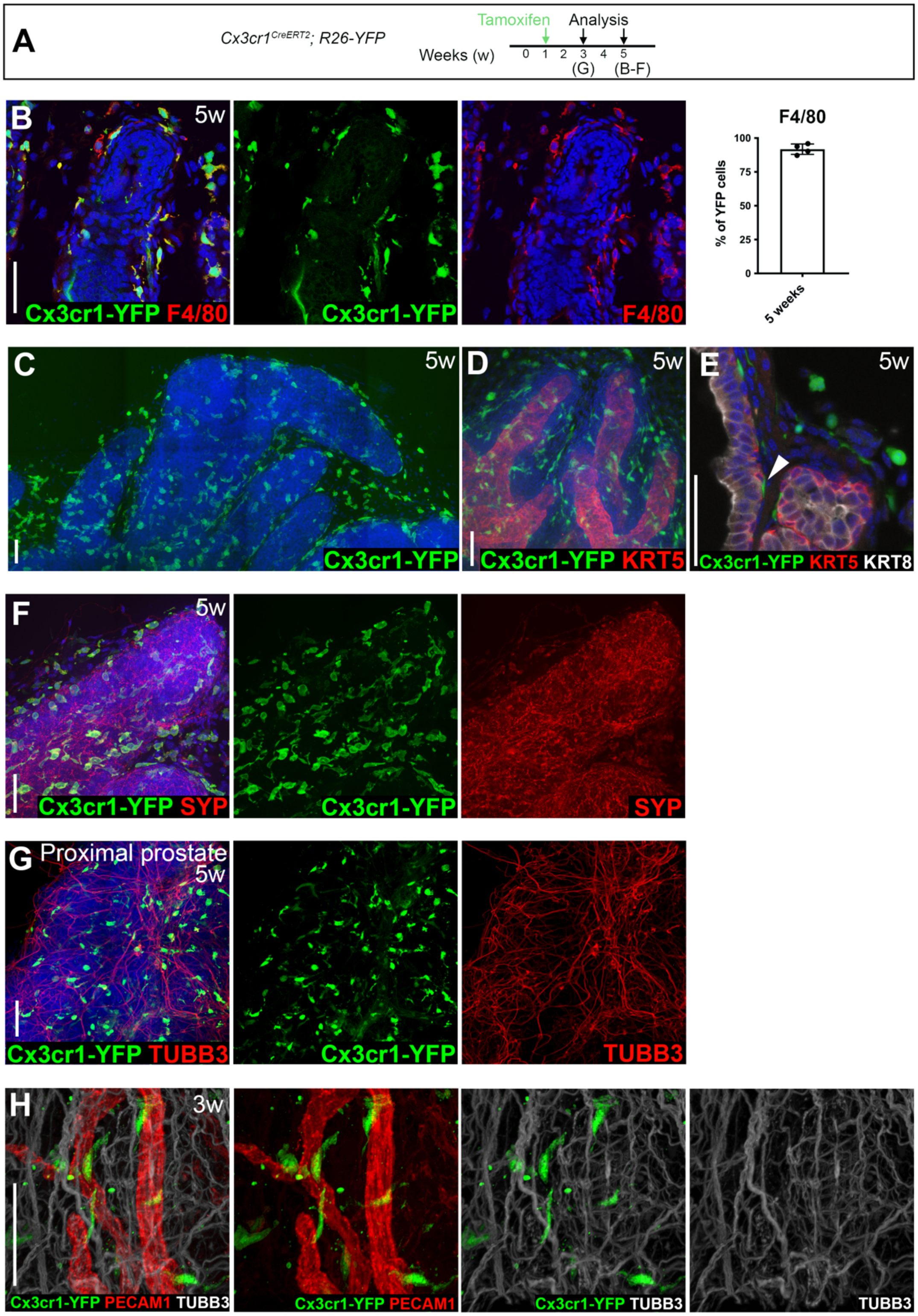

Development • Supplementary information

Fig. S3. Association of prostate macrophages with neurons and vasculature. (A) Timeline for YFP labeling and analysis of anterior prostates (AP) from *Cx3cr1^{CreERT2}; R26-YFP* (Cx3cr1-YFP) mice at 3 to 5-weeks of age. (B) Analysis of tissue sections for F4/80 expression in Cx3cr1-YFP cells and quantitation (n=4 mice, 240 cells). (C) Three-dimensional (3-D) imaging of prostate tissues stained for YFP. (D-J) Immunostaining for YFP and (D) basal cell marker KRT5, (E) KRT5 and luminal cell marker KRT8, (F) neuronal markers synaptophysin (SYP) and (F,G) tubulin beta 3 class III (TUBB3), and (H) endothelial cell marker PECAM1. E is an image of a stained tissue section. Max intensity projections of confocal imaging of (C) 80 µm, (D) 100 µm, (F) 70 µm, (G) 50 µm and (H) two-photon 35 µm Z-stack thickness are shown. Samples sizes were n>2 mice for each staining. Nuclei were stained with DAPI. Scale bars indicate 50 µm. Related to Fig. 3 and Movie 3.

Development • Supplementary information

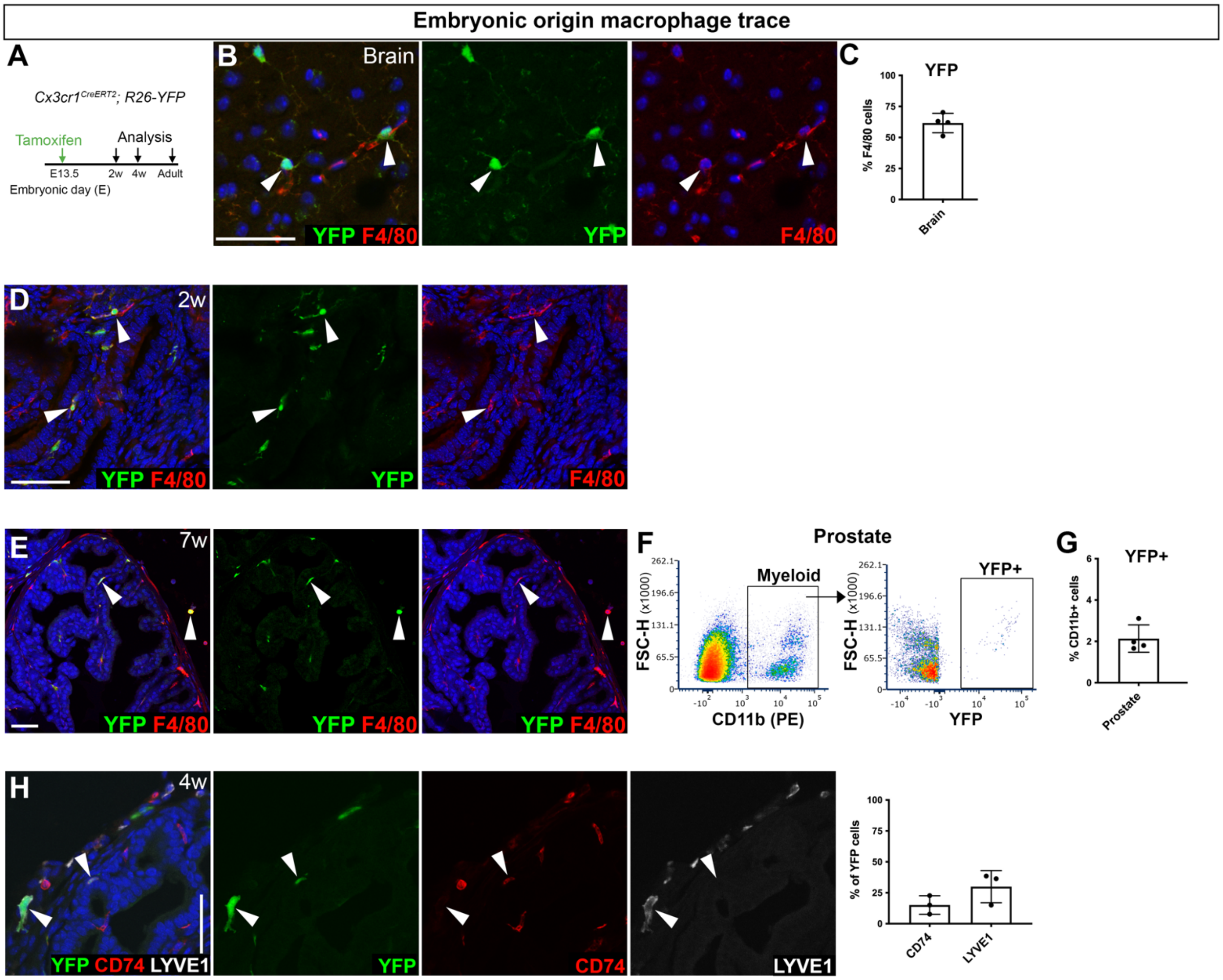

Fig. S4. Lineage-tracing of embryonic origin macrophages in the prostate. (A) Timeline for YFP labeling and analysis of embryonic origin macrophages using *Cx3cr1*^{CreERT2}*; R26-YFP* (Cx3cr1-YFP) mice. (B,C) Analysis of YFP+ microglia in the brain cortex of adult mice after lineage tracing (n=4 mice). (D, E) Immunostaining for Cx3cr1-YFP and macrophage marker F4/80 expression in anterior prostates from mice at (D) 2 weeks and (E) 7 weeks of age. (E-G) Images and quantitation of YFP+ cells in prostates of mice at 7 weeks of age. Arrowheads in D, E point to YFP+ F4/80+ macrophages. (F,G) Flow cytometry analysis and quantitation of CD45+ CD11b+ YFP+ cells from dissociated prostates (n=4 mice). Cells shown were gated on live single CD45+ cells. (H) Immunostaining for YFP, CD74 and LYVE1 expression in prostates from mice at 4 weeks of age and quantitation of the percentage of YFP cells expressing CD74 or LYVE1. Arrowheads point to YFP+ CD74+ and YFP+ LYVE1+ cells (n=3 mice, 55 cells). Nuclei were stained with DAPI. Scale bars indicate 50 µm. Related to Fig. 4.

Development • Supplementary information

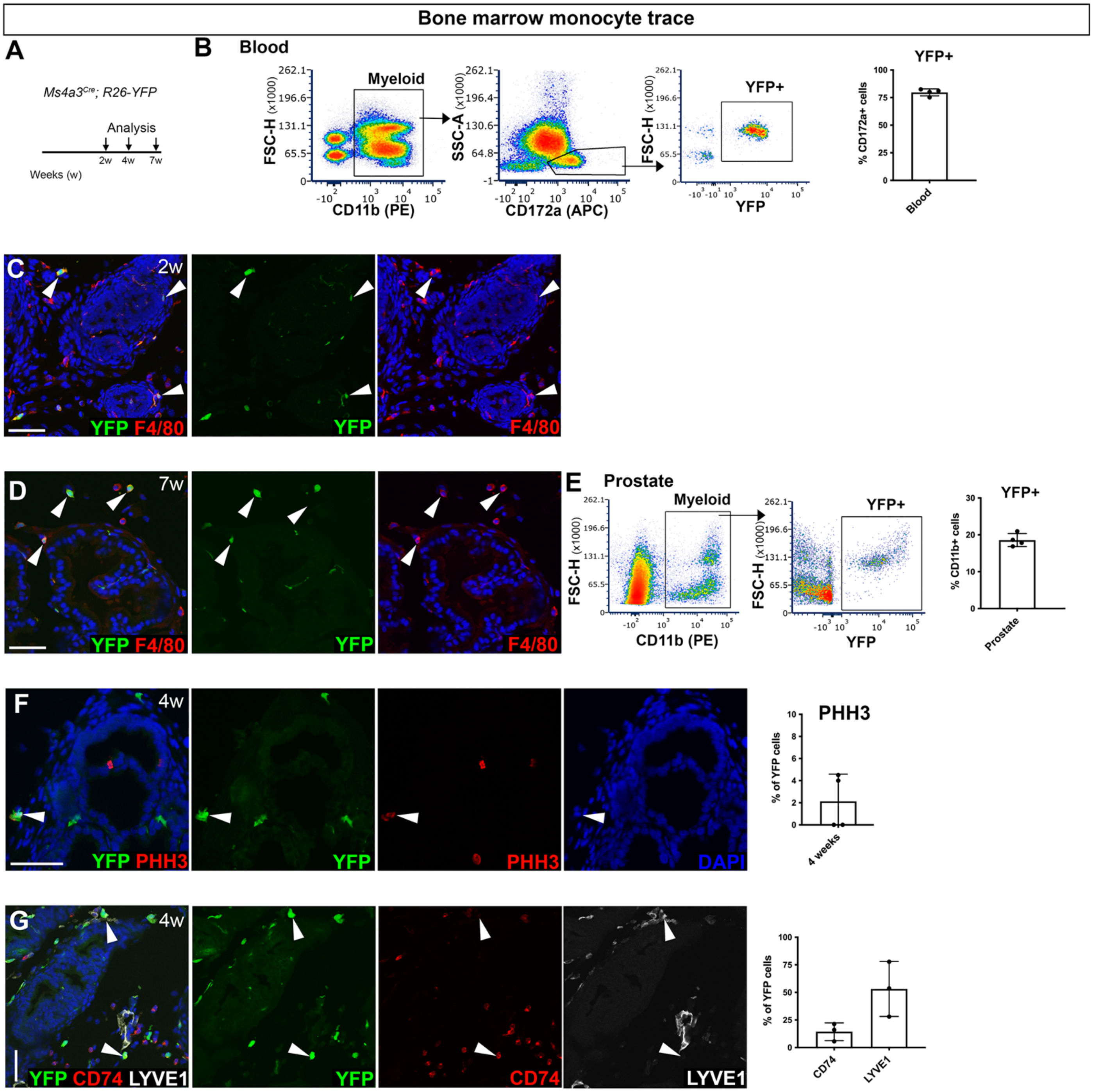

Development • Supplementary information

Fig. S5. Lineage-tracing of bone marrow monocyte-derived cells in the prostate. (A) Timeline for analysis of bone marrow monocyte traced cells in *Ms4a3^{Cre}; R26-YFP* (Ms4a3-YFP) mice. (B) Flow cytometry analysis and quantitation of YFP expression in monocytes from blood. Live cells were gated for CD45+, CD11b+, CD172+ and SSC-A low blood monocytes to determine the percentage of YFP+ blood monocytes (n=4 mice). (C,D) Immunostaining for YFP and F4/80 expression. Prostates from mice at (C) 2 weeks and (D) 7 weeks of age are shown. (E) Flow cytometry analysis of YFP expression in Ms4a3-YFP prostates at 7 weeks of age (n=4 mice). Cells shown were gated on live single CD45+ cells. (F) Immunostaining for YFP and proliferation marker phospho-Histone3 (PHH3; n=4 mice, 120 cells). (G) Immunostaining for YFP, CD74, and LYVE1 expression in prostates at 4 weeks of age and quantitation of the percentage of YFP cells expressing CD74 or LYVE1. Arrowheads point to YFP+ CD74+ and YFP+ Lyve1+ cells. (n=3 mice, 71 cells). Nuclei were stained with DAPI. Scale bars indicate 50 µm. Related to Fig. 4.

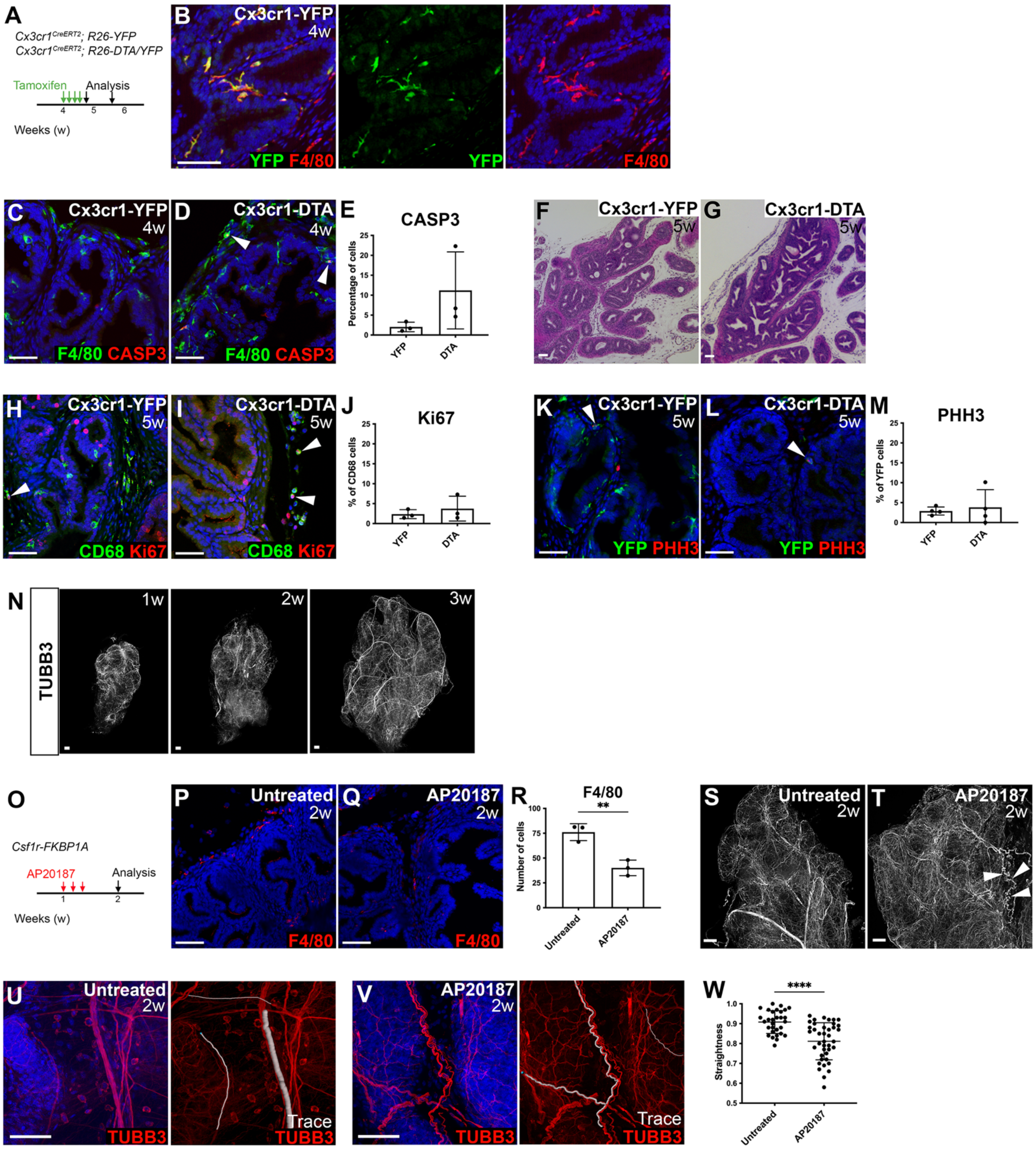

Development • Supplementary information

Fig. S6. Transient ablation of prostate macrophages affects nerve fibers. (A) Timeline for analysis of anterior prostates from *Cx3cr1*^{CreERT2}*; R26-YFP/+* (Cx3cr1-YFP) and *Cx3cr1*^{CreERT2}*; R26-DTA/YFP* (Cx3cr1-DTA) mice. (B-E) Immunostaining for YFP and (B) F4/80 and (C-E) active caspase3 (CASP3) expression one day after tamoxifen treatment (n=3 mice for each group). (F, G) Representative hematoxylin and eosin (H+E) staining of the Cx3cr1-YFP and Cx3cr1-DTA prostates one week after treatment (n=4 mice for each group). (H-M) Immunostaining and quantitative analysis of (H-J) CD68 and Ki67 and (K-M) YFP and phospho-Histone3 (PHH3) expression. (N) Maximum intensity projections of 3-D whole-mount confocal imaging for neuronal marker TUBB3. Anterior prostate lobes from mice at ages 1 week, 2 weeks and 3 weeks are shown. Sample sizes were n=2 mice for each age. (O-W) Analysis of neuronal marker TUBB3 expression after partial ablation of macrophages. (O) Timeline for AP20187 treatment and analysis of anterior prostates from *Csf1r-FKBP1A* mice. (P-R) Analysis and quantitation of F4/80 macrophages in untreated and AP20187 dimerizer-treated mice. (S-V) Maximum intensity projections of 3-D whole-mount confocal imaging of 2 week anterior prostate lobes for neuronal marker TUBB3 in (S,U) control untreated and (T, V) mice treated with AP20187 to induce apoptosis of macrophages. Sample sizes were n=3 mice for each treatment group. (U-W) Nerve fiber tracing (white) of TUBB3 performed in Imaris and (W) quantification of nerve fiber tortuosity. The graph in W represents individual traced nerve fibers, with 10 nerve fibers each from n=3 mice for each treatment group. A value of 1 indicates a straight fiber. Maximum projections are shown of confocal Z-stacks (S-T) 160 µm, and (U,V) 10 µm in thickness. Nuclei were stained with DAPI. Scale bars indicate 50 µm. *P* values were calculated using two-tailed student t-tests. $^{**}p < 0.01$, $^{****}p < 0.0001$. Related to Fig. 5.

Development • Supplementary information

Table S1. Primers for mouse genotyping

Allele	Forward	Reverse
CreERT2	CAGATGGCGCGGCAACACC	GCG CGG TCT GGC AGT AAA AAC
R26-YFP	ACA TGG TCC TGC TGG AGT TC	GGC ATT AAA GCA GCG TAT CC
R26-YFP WT	AAG GGA GCT GCA GTG GAG TA	CCG AAA ATC TGT GGG AAG TC
R26-DTA	CGA CCT GCA GGT CCT CG	CTC GAG TTT GTC CAA TTA TGT CAC
R26-DTA WT	CCA AAG TCG CTC TGA GTT GTT ATC	GAG CGG GAG AAA TGG ATA TG
$Cx3cr1^{CreERT2}$	GTT AAT GAC CTG CAG CCA AG	ACG CCC AGA CTA ATG GTG AC
Cx3cr1 WT	AGC TCA CGA CTG CCT TCT TC	ACG CCC AGA CTA ATG GTG AC
GFP	AAG TTC ATC TGC ACC ACC G	TCC TTG AAG AAG ATG GTG CG
GFP WT	CTA GGC CAC AGA ATT GAA AGA TCT	GTA GGT GGA AAT TCT AGC ATC ATC C
$Ms4a3^{Cre}$	AGA GAA ATC ATC AGG GCA GAA AT	TTG GCG AGA GGG GAA AGA C
$Ms4a3^{Cre}$ WT	AGA GAA ATC ATC AGG GCA GAA AT	GAA AGG GGA ACA AGC GAA GAT

Table S2. Antibodies

Primary antibodies for immunofluorescence			
**Antigen**	**Supplier**	**Species**	**Dilution**
Active Caspase 3	BD Biosciences 559565	rabbit IgG	1:200
AR	Abcam ab133273	rabbit IgG	1:100
CD68	Boster PA1518	rabbit IgG	1:250
CD74	R&D Systems MAB7478	rat IgG2a	1:500
KRT5	Covance PRB-160P	rabbit IgG	1:500
KRT5	Covance SIG-3475	chicken IgY	1:500
KRT8/18	Dev studies hybridoma bank TROMA-I	rat IgG2a	1:100
F4/80	Biorad MCA497RT	rat IgG2b	1:200
Ki67	eBiosciences 14-5698-80	rat IgG2a	1:1000
GFP/YFP	Abcam ab13970	chicken IgY	1:1000
GFP/YFP	Cell Signaling 2956	rabbit IgG	1:1000
LYVE1	Abcam ab14917	rabbit IgG	1:500
PECAM1 (CD31)	BD Biosciences 550274	rat IgG2a	1:200
p-Histone3 (Ser10)	Invitrogen MA5-15220	mouse IgG1	1:250
Synaptophysin	BD 611880	mouse IgG1	1:300
TUBB3 (TuJ-1)	R&D Systems MAB1195	mouse IgG2A	1:500

Secondary antibodies for immunofluorescence		
**Antibody**	**Supplier**	**Dilution**
goat anti-chicken Alexa Fluor 488	Invitrogen A11039	1:500
goat anti-chicken Alexa Fluor 555	Invitrogen A21437	1:500
goat anti-mouse Alexa Fluor 555	Invitrogen A21424	1:500
goat anti-mouse CF640R	Biotium 20304	1:500
goat anti-rabbit Alexa Fluor 488	Invitrogen A11008	1:500
goat anti-rabbit Alexa Fluor 555	Invitrogen A21428	1:500
goat anti-rat Alexa Fluor 555	Invitrogen A21434	1:500
goat anti-rat Alexa Fluor 633	Invitrogen A21094	1:500

Antibodies for flow cytometry		
**Antigen**	**Supplier**	**Dilution**
CD11b-PE (clone M1/70)	Biolegend 101207	1:100
CD11c-APC/Cyanine7 (clone N418)	Biolegend 117323	1:100
CD45.2-Alexa Fluor 700 (clone 104)	Biolegend 109821	1:100
CD172a-APC (SIRPα) (clone P84)	Biolegend 144013	1:100
F4/80-Pacific Blue (clone BM8)	Biolegend 123123	1:50
Ly6G-PerCP/Cyanine 5.5 (clone 1A8)	Biolegend 127615	1:100
LIVE/DEAD Fixable Aqua	Invitrogen L34965	1:1000

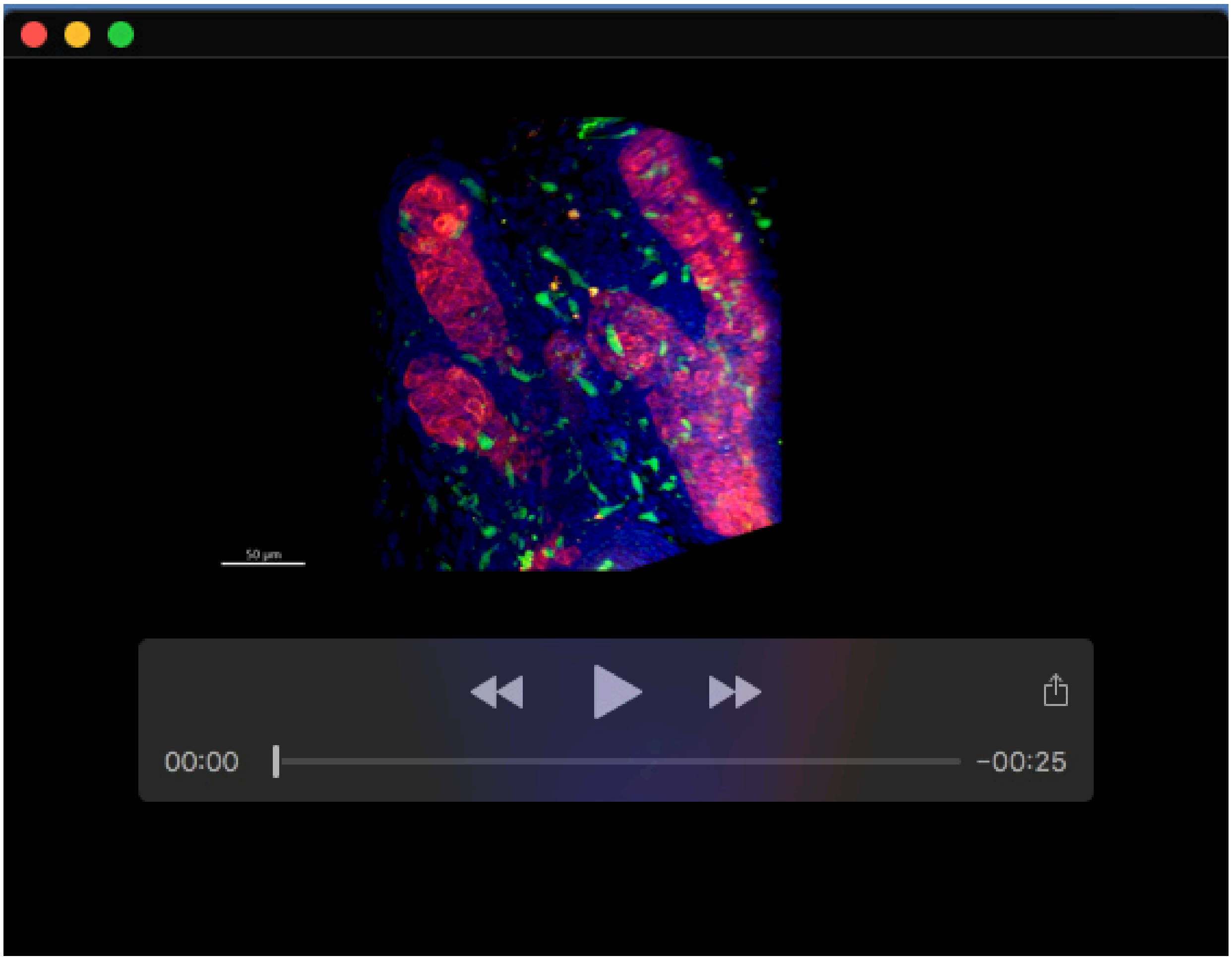

Movie 1. Cx3cr1-YFP macrophages surround the prostate duct and can associate with basal cells. 3-D rendering of a 50µm Z-stack confocal image of the distal tips of an anterior prostate (AP) at 5 weeks of age. Cx3cr1-YFP (green) and immunolabeling for KRT5 (red) are shown. Nuclei were stained with DAPI (blue). Surfaces created using Imaris software were pseudo-colored with the same scheme, and Cx3cr1-YFP cells in contact with KRT5 expressing cells are colored in aqua. Related to Fig. 3.

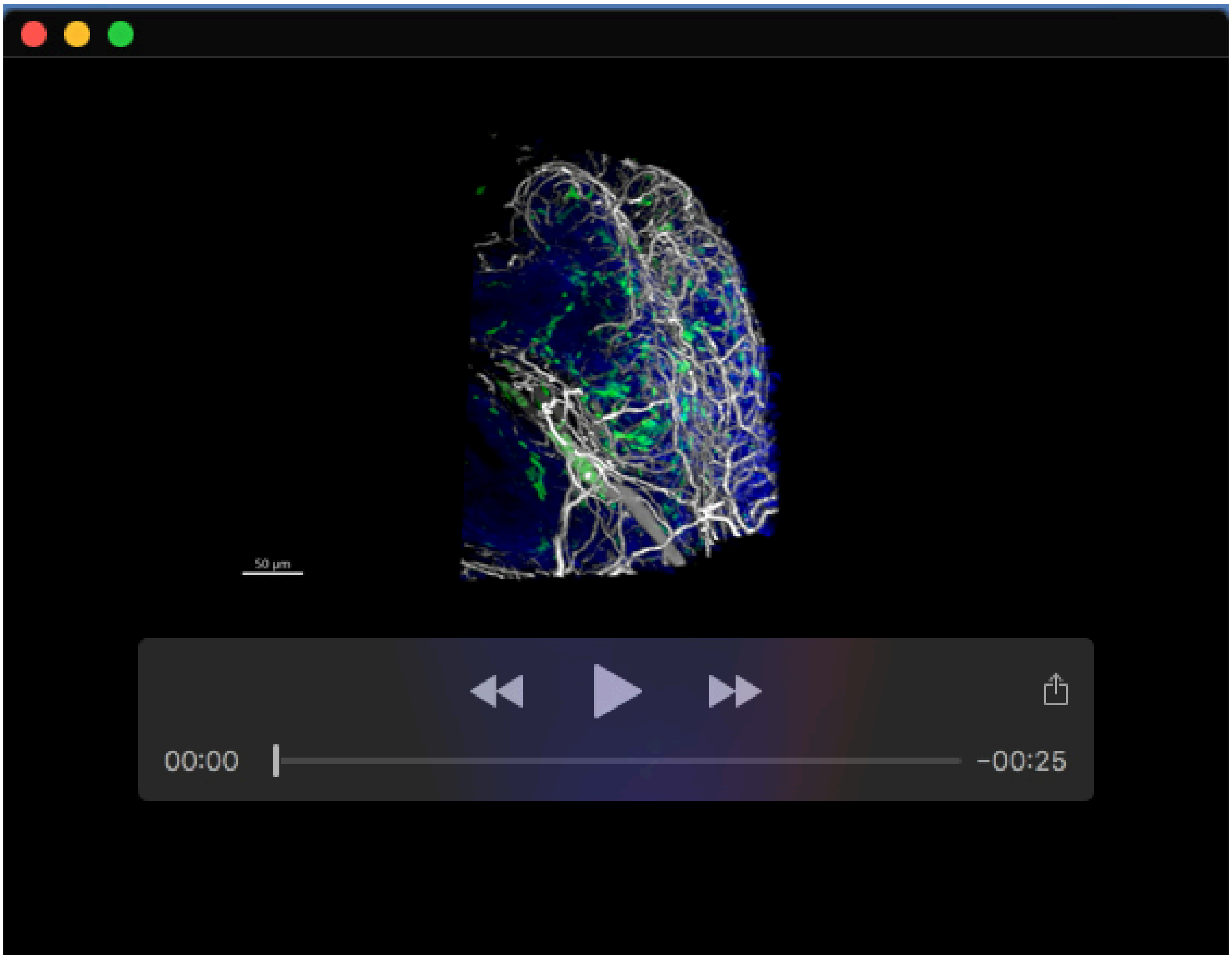

Movie 2. Cx3cr1-YFP macrophages associate with neurons at the surface of the prostate duct. 3-D rendering of a 100µm Z-stack confocal image of the distal tips of an anterior prostate (AP) at 5 weeks of age. Cx3cr1-YFP (green) and immunolabeling for TUBB3 (white) are shown. Nuclei were stained with DAPI (blue). Surfaces were created using Imaris software were pseudo-colored with the same scheme. Related to Fig. 3 and Fig. 5.

Development • Supplementary information

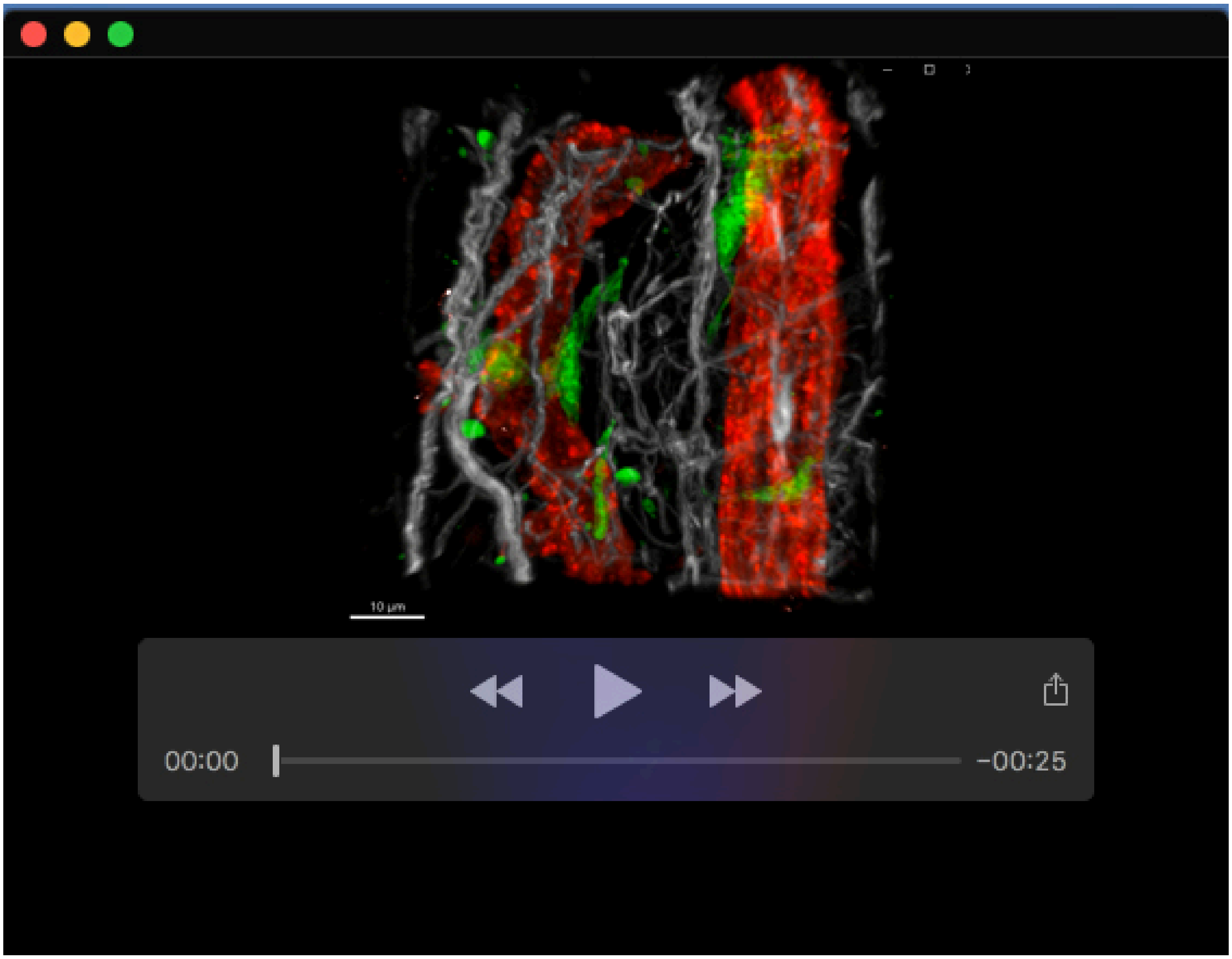

Movie 3. Cx3cr1-YFP macrophages are in close proximity to nerve fibers and blood vessels in the prostate. 3-D rendering of a 35µm Z-stack two-photon spectral image at the surface of the anterior prostate (AP) at 3 weeks of age. Cx3cr1-YFP (green) and immunolabeling for CD31 (red) and TUBB3 (white) are shown. Maximum projection 3-D rendering was created using Imaris software. Related to Fig. 3.
